# *Aggregatibacter aphrophilus *in a patient with recurrent empyema: a case report

**DOI:** 10.1186/1752-1947-5-448

**Published:** 2011-09-12

**Authors:** Lasantha Ratnayake, William J Olver, Tom Fardon

**Affiliations:** 1 Department of Medical Microbiology, Level 6, Ninewells Hospital, Dundee, DD1 9SY, UK; 2 Department of Respiratory Medicine, Ninewells Hospital, Dundee, DD1 9SY, UK

## Abstract

**Introduction:**

*Aggregatibacter aphrophilus *(formerly *Haemophilus aphrophilus *and *H. paraphrophilus*) is classically associated with infective endocarditis. Other infections reported in the literature include brain abscess, bone and joint infections and endophthalmitis. There are only two cases of empyema ever reported due to this organism. We report the isolation of *A. aphrophilus *from pleural fluid on three separate hospital admissions in a patient with recurrent empyema.

**Case presentation:**

A 65-year-old female patient of Caucasian origin presented with a three-week history of fever, shortness of breath and dry cough. She was found to have a pleural empyema so a chest drain was inserted and a sample of pus was sent to the microbiology laboratory. After overnight incubation, a chocolate blood agar plate incubated in 5% carbon dioxide showed a profuse growth of small, round, glistening colonies which were identified as Gram-negative coccobacilli. They were oxidase- and catalase-negative. Biochemical testing using RapID NH confirmed the identity of the organism as *A. aphrophilus*. It was susceptible to amoxicillin, levofloxacin and doxycycline. Our patient was treated with intravenous amoxicillin with clavulanic acid and clarithromycin followed by oral doxycycline, but was re-admitted twice over the next three months with recurrent empyema and the same organism was isolated. Each episode was managed with chest drainage and a six-week course of antibiotic--doxycycline for the second episode and amoxicillin for the third episode, after which she has remained well.

**Conclusion:**

This is the first case report of recurrent empyema due to *A. aphrophilus*. Our patient had no underlying condition to explain the recurrence. Although our isolate was doxycycline susceptible, our patient had recurrent infection after treatment with this antibiotic, suggesting that this antibiotic is ineffective in treatment of deep-seated *A. aphrophilus *infection. This organism can be difficult to identify in the laboratory because, unlike closely related *Haemophilus spp*., it is oxidase-negative, catalase-negative and X and V independent.

## Introduction

*Aggregatibacter aphrophilus *(formerly *Haemophilus aphrophilus *and *H. paraphrophilus*) is part of the normal oropharyngeal flora. It is a Gram-negative coccobacillus that requires 5% carbon dioxide (CO_2_) for primary isolation, growing best on chocolate blood agar. It can be difficult to identify in the laboratory because, unlike closely related *Haemophilus spp.*, it is oxidase-negative, catalase-negative and X and V independent. It was first described by Khairat in 1940 when it was isolated from a patient with infective endocarditis [1]. He chose the species name to reflect the requirement for CO_2 _(literally 'froth-loving'). Other infections reported in the literature include brain abscess, bone and joint infections and endophthalmitis [2]. Empyema was first described in 1965 in a patient who responded to a combination of penicillin and tetracycline [3]. A second case in more recent times was treated with amoxicillin with clavulanic acid [2]. Antibiotic therapy with amoxicillin +/- beta-lactamase inhibitor, third generation cephalosporins or fluoroquinolones have all been used successfully to treat *A. aphrophilus *infections [2]. However, resistance to cephalosporins has been described [4]. We report for the first time the isolation of *A. aphrophilus *from pleural fluid on three separate hospital admissions in a patient with recurrent empyema.

## Case presentation

A 65-year-old female patient of Caucasian origin was admitted to our hospital with a three-week history of fever, shortness of breath and dry cough. She did not complain of hemoptysis or loss of weight, she was not a smoker and she had no history of underlying lung disorders. On examination her temperature was 37.9°C. Her respiratory rate was 20 breaths per minute with an oxygen saturation of 88% on air. An examination of her respiratory system revealed decreased breath sounds and dullness to percussion in the base of her right lung. Examination of her cardiovascular system, abdomen and central nervous system were normal. Investigations showed a white cell count of 28.2 × 10^9^/L (normal range 4-11 × 10^9^/L) with neutrophilia, and her C-reactive protein level was 429 mg/L (normal range up to 5 mg/L).

**Figure 1:**
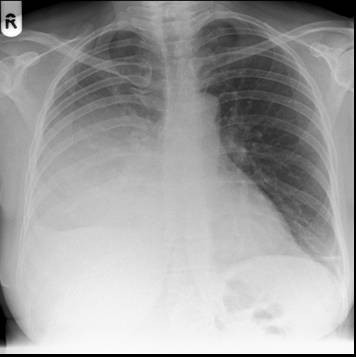
**Chest X-ray on admission**. This X-ray image shows a large right-sided pleural effusion.

**Figure 2:**
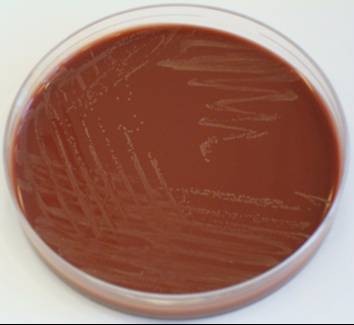
**Growth on chocolate agar**. This shows a profuse growth of small, round, glistening colonies.

A chest X-ray on admission showed opacification of her right middle and lower zone and moderate right-sided pleural effusion (Additional file 1 and Figure 1). She was started on intravenous amoxicillin with clavulanic acid and clarithromycin as per hospital protocol for severe community-acquired pneumonia. A wide bore chest drain was inserted and a sample of pus was inoculated into aerobic and anaerobic blood culture bottles. On day two of incubation in the BacT/ALERT automated blood culture system (bioMérieux, Basingstoke, UK) both bottles signaled positive but no organisms were seen on Gram stain. After overnight incubation at 37°C there was poor growth on blood agar, but the chocolate blood agar plate incubated in 5% CO_2 _showed a profuse growth of small, round, glistening colonies (Additional file 1 and Figure 2) which were identified as Gram-negative coccobacilli. Biochemical panel testing on our isolate using API NH (bioMérieux, Basingstoke, UK) was inconclusive but *A. aphrophilus *was suspected because it was oxidase-negative, catalase-negative and × and V factor independent. Identification of our isolate was confirmed by the reference laboratory using RapID NH (Oxoid, Basingstoke, UK). Our patient responded to intravenous amoxicillin with clavulanic acid and clarithromycin followed by oral doxycycline (two weeks total antibiotic course). However she was readmitted twice over the next three months with recurrent empyema and the same organism was isolated. Each episode was managed with chest drainage and a six-week course of antibiotics; doxycycline for the second episode and amoxicillin for the third, after which she has remained well.

## Discussion

*A. aphrophilus *is the species which now includes both V factor independent (formerly *H. aphrophilus*) and V factor dependent (formerly *H. paraphrophilus*) strains. Both are × factor independent, although the former requires hemin-containing media on primary isolation. It is oxidase-negative and catalase-negative, in contrast to the more commonly-isolated *H. influenzae *and *H. parainfluenzae*, which are both oxidase- and catalase-positive. Because of this, diagnostic laboratories may have difficulty in identifying *A. aphrophilus*. Indeed, there are reports of this organism being misidentified as *Pasteurella spp*. [5,6].

This case report highlights a very unusual presentation of *A. aphrophilus*, which is more commonly associated with infective endocarditis. Our patient was investigated for possible underlying causes of recurrent empyema. Factors predisposing to aspiration, such as altered mental status, alcoholism and periodontal disease have been linked to the development of empyema. However none of these were applicable to our patient. There was no evidence of infective endocarditis, malignancy, lung abscess, subdiaphragmatic infection or esophageal leak and no history of thoracic trauma or surgery. Her immunoglobulin levels were normal. Despite two courses of doxycycline our patient's empyema recurred, but it was successfully treated with amoxicillin on the third episode. Despite the organism's susceptibility to doxycycline, our experience suggests that this antibiotic is ineffective in treatment of deep-seated *A. aphrophilus *infections. Although the 1965 case report patient was successfully treated with tetracycline, it was in combination with high-dose penicillin. In more recent times amoxicillin +/- beta-lactamase inhibitor, third-generation cephalosporins and fluoroquinolones have all been used to successfully treat these infections [2].

Unfortunately there are no conclusive studies on duration of antibiotic therapy for most bacterial pleural space infections. The British Thoracic Society has published guidelines on managing pleural infections and recommends three weeks of antibiotic therapy [7]. The treatment of the first episode of infection in our patient could therefore be considered inadequate as she only had two weeks of antibiotics.

## Conclusion

To the best of our knowledge, this is the first case report of recurrent empyema due to *A. aphrophilus*. Our patient had no underlying condition to explain recurrent empyema. Although our isolate was doxycycline susceptible, our patient had recurrent infection after treatment with this antibiotic, suggesting that this antibiotic is ineffective in treatment of deep-seated *A. aphrophilus *infection. This organism can be difficult to identify in the laboratory because, unlike closely related *Haemophilus spp.*, it is oxidase-negative, catalase-negative and X and V independent.

## Consent

Written informed consent was obtained from the patient for publication of this case report and any accompanying images. A copy of the written consent is available for review by the Editor-in-Chief of this journal.

## Competing interests

The authors declare that they have no competing interests.

## Authors' contributions

LR and WO identified the organism in the laboratory, gave advice on antibiotic management and prepared the manuscript. TF was the patient's physician and contributed to the manuscript. All authors read and approved the final manuscript.

## Supplementary Material

Additional file 1 **Haemophilus images**. Click here for file
