# Supplementary figures and images for: Aggregatibacter aphrophilus in a patient with recurrent empyema: a case report
Source: J Med Case Rep. 2011 Sep 12;5:448. doi: 10.1186/1752-1947-5-448 (PMC3177941; doi:10.1186/1752-1947-5-448)

## Slide 1
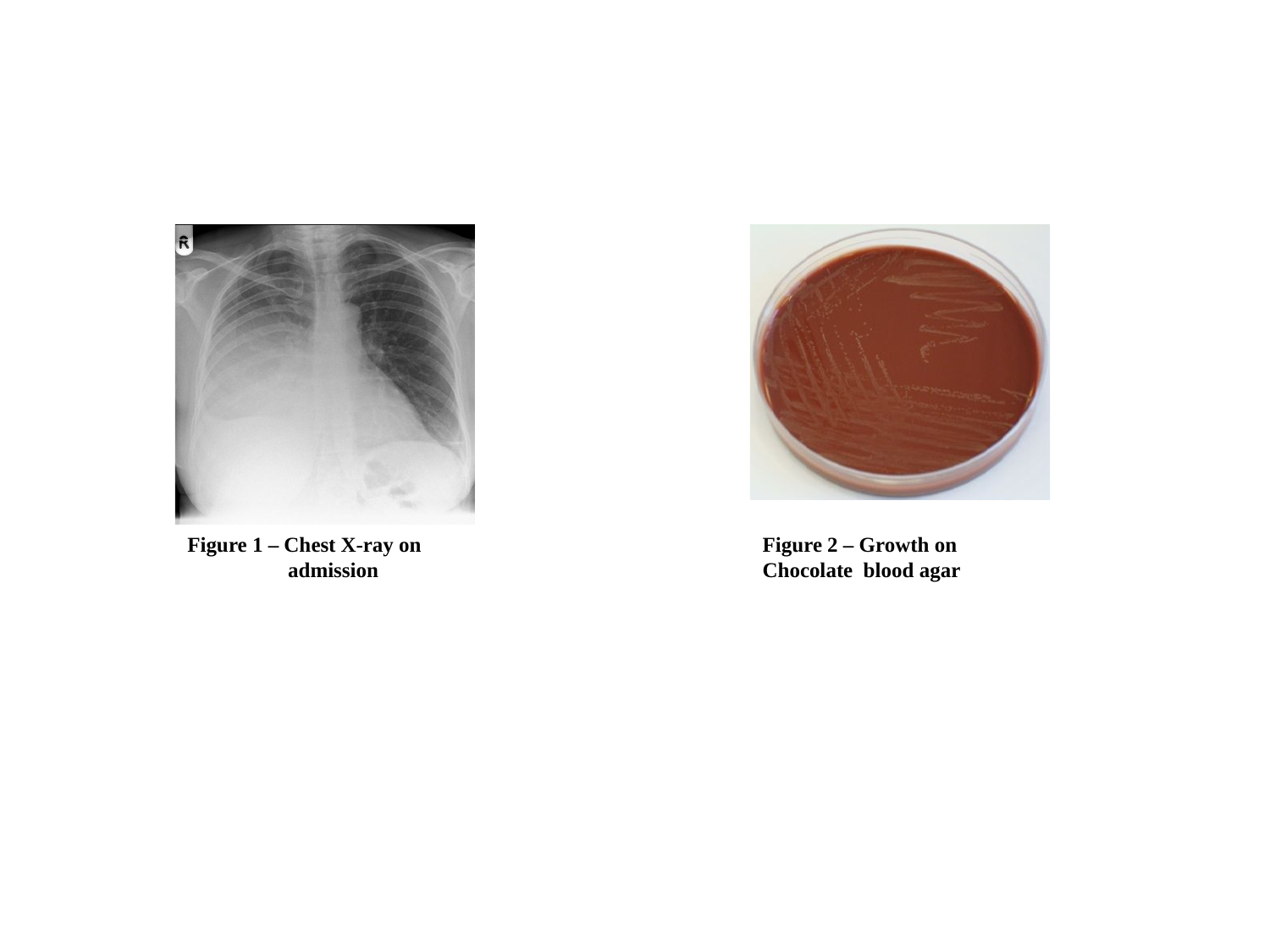

#
Figure 1 – Chest X-ray on admission
Figure 2 – Growth on Chocolate blood agar

Supplement: Additional file 1 — Haemophilus images. [file 1752-1947-5-448-S1.PPT]
